# A g-C_3_N_4_/rGO/Cs_3_Bi_2_Br_9_ mediated Z-scheme heterojunction for enhanced photocatalytic CO_2_ reduction†

**DOI:** 10.1039/d4ta01857e

**Published:** 2024-05-28

**Authors:** Yasmine Baghdadi, Matyas Daboczi, Filipp Temerov, Mengya Yang, Junyi Cui, Salvador Eslava

**Affiliations:** a Department of Chemical Engineering and Centre for Processable Electronics, Imperial College London London SW7 2AZ UK s.eslava@imperial.ac.uk; b Nano and Molecular System (NANOMO) Research Unit, University of Oulu Oulu 90570 Finland

## Abstract

Photocatalytic CO_2_ reduction plays a crucial role in advancing solar fuels, and enhancing the efficiency of the chosen photocatalysts is essential for sustainable energy production. This study demonstrates advancements in the performance of g-C_3_N_4_ as a photocatalyst achieved through surface modifications such as exfoliation to increase surface area and surface oxidation for improved charge separation. We also introduce reduced graphene oxide (rGO) in various ratios to both bulk and exfoliated g-C_3_N_4_, which effectively mitigates charge recombination and establishes an optimal ratio for enhanced efficiency. g-C_3_N_4_/rGO serves to fabricate a hybrid organic/inorganic heterojunction with Cs_3_Bi_2_Br_9_, resulting in a g-C_3_N_4_/rGO/Cs_3_Bi_2_Br_9_ composite. This leads to a remarkable increase in photocatalytic conversion of CO_2_ and H_2_O to CO, H_2_ and CH_4_ at rates of 54.3 (±2.0) μmol_e^−^_ g^−1^ h^−1^, surpassing that of pure Cs_3_Bi_2_Br_9_ (11.2 ± 0.4 μmol_e^−^_ g^−1^ h^−1^) and bulk g-C_3_N_4_ (5.5 ± 0.5 μmol_e^−^_ g^−1^ h^−1^). The experimentally determined energy diagram indicates that rGO acts as a solid redox mediator between g-C_3_N_4_ and Cs_3_Bi_2_Br_9_ in a Z-scheme heterojunction configuration, ensuring that the semiconductor (Cs_3_Bi_2_Br_9_) with the shallowest conduction band drives the reduction and the one with the deepest valence band (g-C_3_N_4_) drives the oxidation. The successful formation of this high-performance heterojunction underscores the potential of the developed composite as a photocatalyst for CO_2_ reduction, offering promising prospects for advancing the field of solar fuels and achieving sustainable energy goals.

## Abstract 2

A hybrid Z-scheme composite structure made of exfoliated g-C_3_N_4_ and Cs_3_Bi_2_Br_9_ semiconductors and a reduced graphene oxide redox mediator is presented for enhanced photocatalytic CO_2_ and H_2_O conversion to CO, CH_4_, and H_2_.

## Graphical abstract

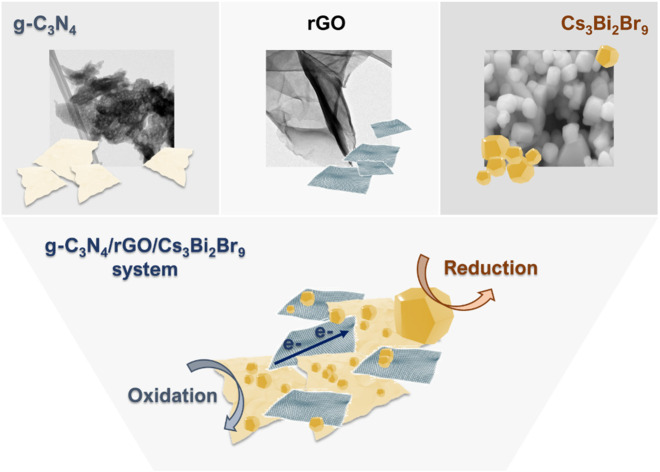

## Introduction

The photocatalytic reduction of carbon dioxide (CO_2_) is critically important in addressing future environmental and energy challenges. This innovative method leverages solar energy to convert CO_2_ into valuable hydrocarbons and fuels, thereby mitigating greenhouse gas emissions and combatting climate change.^1,2^ Utilizing solar energy photocatalysis presents a sustainable and efficient avenue for transforming CO_2_ into useful products, thus reducing reliance on fossil fuels and contributing to a greener, more carbon-neutral future. The outcome of photocatalytic redox reactions is influenced by numerous factors, including the optoelectronic properties of the selected photocatalyst and the effective separation of photo-induced charges.^3^

Over the past decade, a variety of photocatalytic materials, such as TiO_2_, SrO_2_, WO_3_, and CdS, have been tested under a variety of reaction conditions, such as in photocatalytic water treatment, dye degradation, CO_2_ reduction, or water splitting.^4–8^ Halide perovskites attract attention due to their long carrier diffusion lengths, tunable bandgaps, good quantum efficiency, and suitable absorption coefficients.^9,10^ Among these perovskites, Cs_3_Bi_2_Br_9_ (CBB) has recently emerged as a promising candidate for CO_2_ reduction reactions due to its advantageous optoelectronic properties, including a shallow conduction band edge that encompasses the redox potential of CO_2_ reduction (CO_2_/CO at −0.53 V *vs.* NHE at pH = 7). CBB consists of a three-dimensional framework made up of cesium (Cs), bismuth (Bi), and bromine (Br) ions, forming a cage-like structure.^11,12^ This arrangement gives CBB its unique perovskite-like structure and optoelectronic properties and offers a non-toxic and stable alternative to lead-based perovskites.^13–15^

To enhance the efficiency of the CO_2_ photocatalytic reduction process, separation of photogenerated charges in a heterojunction with a secondary semiconductor is a promising approach.^9,16,17^ One prospective candidate semiconductor for heterojunction formation is graphitic carbon nitride (GCN). GCN has often been reported as a photocatalyst for water splitting coupled with other semiconductors such as Bi_2_S_3_, TiO_2_, BiOCl, or Al_2_O_3_ to enhance change separation and overall efficiency.^18–21^ GCN is a carbon-based semiconducting material formed through the polymerization condensation of different carbon and nitrogen rich monomers such as urea, melamine, or dicyanamide.^22,23^ Within the structure of GCN, the C and N atoms are both sp^2^ hybridized forming a hexagonal triazine rings connected by the N atoms.^24^ GCN is well known for its high chemical and thermal stability and for its straightforward and inexpensive synthesis. The structure of GCN allows electronic tuning of valence and conduction bands which are appropriately situated for photocatalytic water splitting and CO_2_ reduction.^25,26^

Photocatalytic heterojunctions between CBB and graphitic carbon nitride (GCN) combine inorganic and organic materials and the unique properties of both components for enhanced photocatalytic activity, such as the visible-light response and excellent stability of GCN. In a previous study, we achieved a GCN/CBB composite that converts CO_2_ and H_2_O vapors to CO (14.2 μmol g^−1^ h^−1^) under ambient conditions of temperature and pressure. The enhanced production was due to the formation of a direct Z-scheme configuration where photogenerated charges in the semiconductors were spatially separated, keeping CBB as the reduction site and GCN as the oxidation site.^15^

Reduced graphene oxide (rGO), characterized by its sheet-like two-dimensional structure and electrical conductivity close to that of graphene, has emerged as a material of interest. Despite having structural defects and residual functional groups from the reduction process of graphene oxide, rGO is used in various applications, including electronics, energy storage devices, and as a redox mediator in Z-scheme photocatalytic systems. Its high electrical conductivity and ability to promote efficient charge transfer make it an ideal candidate to enhance the overall photocatalytic activity of a system.^27–30^

In this study, we introduce GCN/rGO/CBB composites designed for enhanced conversion of CO_2_ and H_2_O(g) into CO, CH_4_ and H_2_. The performance of these composites (54.3 ± 2.0 μmol_e^−^_ g^−1^ h^−1^ on an electron basis) significantly surpasses that of pure bulk GCN (5.5 ± 0.5 μmol_e^−^_ g^−1^ h^−1^) and pure CBB (11.2 ± 0.4 μmol_e^−^_ g^−1^ h^−1^). To achieve this, we first improved GCN through exfoliation and surface oxidation, enhancing its surface area and the lifespan of its photoinduced charges. We then incorporated rGO sheets to suppress charge recombination by transporting electrons away from GCN. Finally, we constructed a complete heterojunction system with CBB, which further enhanced the separation of the photogenerated charge carriers leading to significantly boosted photocatalytic activity. This study not only highlights the impact of exfoliation and surface oxidation on GCN's performance but also emphasizes the importance of optimizing rGO ratios to suppress charge recombination while preventing the obstruction of light absorption by semiconductors. The successful development of this high-performance heterojunction underscores the potential of the resultant composite as an efficient photocatalyst for CO_2_ reduction, paving the way for new avenues in sustainable energy conversion.

## Experimental

### Materials

For the syntheses, the following materials were used as received: CsBr (99%, Sigma-Aldrich), BiBr_3_ (99%, Sigma-Aldrich), melamine (99%, Alfa Aesar), dimethyl sulfoxide (≥99.9%, Sigma-Aldrich), anhydrous 2-propanol (99.5%, Sigma-Aldrich), ethanol absolute (Sigma-Aldrich), and nitric acid (70%, Sigma-Aldrich). Ethylene glycol (99%, Acros Organics), used for hydrothermal reductions, was also used as received.

### Synthesis of Cs_3_Bi_2_Br_9_ (CBB) crystals

Cs_3_Bi_2_Br_9_ (CBB) crystals were synthesized using an anti-solvent crystallization process. A mixture of 1.2 mmol (207.85 mg) CsBr and 0.8 mmol (292.15 mg) BiBr_3_ was dissolved in 10 mL dimethyl sulfoxide and stirred at room temperature for 2 h at 650 rpm. This solution was then quickly added to a 500 mL round-bottom flask containing 500 mL of isopropanol and stirred vigorously for 1 min. The resulting suspension was centrifuged at 10 000 rpm and washed three times with isopropanol to remove excess dimethyl sulfoxide. The slurry was dried overnight at 40 °C in a vacuum oven and stored in a nitrogen-filled glovebox.

### Synthesis of bulk g-C_3_N_4_ (BGCN)

Bulk g-C_3_N_4_ (BGCN) was obtained by calcining 5 g of melamine in air in a 50 mL covered alumina crucible. The crucible was heated at a rate of 10 °C min^−1^ to 550 °C and the final temperature was maintained for 4 h. After cooling to room temperature, the coarse powder was ground using a mortar and pestle.

### Synthesis of exfoliated g-C_3_N_4_ (EGCN)

Exfoliated g-C_3_N_4_ (EGCN) was synthesized using BGCN as a starting material. 2 g of BGCN was dispersed in 30 mL of nitric acid (70%) and the dispersion was ultrasonicated for different durations (0.5, 2, and 4 h). Subsequently, 150 mL of distilled water was added slowly, and the dispersion was centrifuged at 10 000 rpm and washed five times with ethanol and water to remove nitric acid. The slurry was then dried at 60 °C in a vacuum oven for 12 h. The obtained samples were labeled as #EGCN, where ‘#’ indicates the hours of ultrasonication.

### Synthesis of g-C_3_N_4_/rGO (BGCN/rGO and EGCN/rGO)

Graphene oxide (GO) was prepared following a method previously reported by Eslava *et al.* using a modified Hummer's method.^31^ To synthesize g-C_3_N_4_/rGO, 500 mg of either BGCN or EGCN were dispersed in 40 mL ethylene glycol. A specific amount of GO, to achieve 1, 2.5, or 5 wt% in the final composite, was added to the mixture and ultrasonicated for 1 h. This dispersion was stirred overnight, then added to a 50 mL Teflon-lined autoclave and heated to 180 °C for 24 h. The resulting samples were centrifuged at 10 000 rpm for 10 min in five washing cycles with ethanol and distilled water and then dried at 60 °C in a vacuum oven for 12 h. The samples were denoted as BGCN/#rGO or EGCN/#rGO, where ‘#’ represents the nominal weight percentage of GO.

### Synthesis of composites

To prepare the composites, 250 mg of the desired GCN (BGCN, EGCN, BGCN/rGO, or EGCN/rGO) were dispersed in 500 mL of isopropanol. The mixture was then ultrasonicated for 1 h to ensure homogeneous dispersion. A stoichiometric solution of CsBr and BiBr_3_ in dimethyl sulfoxide was swiftly added to the GCN dispersion and stirred vigorously for 1 min. The GCN content was set at 40 wt% relative to the total sample mass, based on our previous study.^15^ The final composites were labeled as BGCN/rGO/CBB and EGCN/rGO/CBB.

### Characterization

The structural properties of the samples were analyzed using powder X-ray diffraction (XRD), performed on an Xpert Pro PANalytical diffractometer. This equipment operated at a voltage of 40 kV and a current of 40 mA, using Cu Kα radiation (*λ* = 0.15418 nm) over a 2*θ* range of 5–65°. The diffractograms were analyzed using MDI Jade 6 software. Fourier transform infrared spectroscopy (FT-IR) was conducted to study the surface oxidation of organic samples, utilizing an Agilent Technologies Cary 630 spectrometer in attenuated total reflection (ATR) mode. X-ray photoelectron spectroscopy (XPS) was employed to determine the elemental composition of the samples, using a Thermo Fisher K-Alpha+ instrument with a monochromated Al Kα X-ray source. All binding energies were corrected with respect to adventitious carbon at 284.8 eV, and the spectra were processed using Avantage software.

To assess optoelectronic properties, a variety of techniques were used. UV-vis diffuse reflectance spectroscopy (DRS) of the powders was conducted on a Shimadzu UV-3000, equipped with an integrating sphere, using barium sulfate (BaSO_4_) as a reference. The Kubelka–Munk function *F*(*R*) was calculated using the reflectance (*R*) as *F*(*R*) = (1 − *R*)^2^/(2*R*), and Tauc plots of [*F*(*R*)*hν*](1/*n*) *versus* light energy *hν* were plotted to determine using reflectance (*R*) data, where *F*(*R*) = (1 − *R*)^2^/(2*R*), and *hν* represents the light energy and *n* is a constant (0.5 for direct E_g_ and 2 for indirect E_g_). Ambient photoemission spectroscopy (APS), conducted using a KP Technology SKP5050, was employed to measure the cube root photoemission of the samples, with the values extrapolated to zero to ascertain the valence band edge (*E*_v_) values of the thin layers. Monochromatic UV light within the 4.4–6.4 eV range was utilized for irradiation. Kelvin probe measurements documented the contact potential difference between the tip and the sample. A cleaned silver reference was used to calibrate the tip work function, allowing the determination of the Fermi level of the samples by considering the measured contact potential difference and the tip's work function. A uniform thin layer of the powder was measured on indium tin oxide (ITO). The photoluminescence (PL) spectra of the powders were recorded using a photoluminescence spectrometer (FLS1000 Edinburgh Instruments), with the excitation set at 380 nm with a bandwidth of 10 nm, generated using a 450 W ozone-free continuous xenon arc lamp. A long-pass filter with a cut-off wavelength of 395 nm was employed before the detector, and an emission bandwidth of 5 nm, a dwell time of 0.1 s, and a spectral resolution of 1 nm were used.

The morphological properties of the samples were primarily studied using scanning electron microscopy (SEM) on a Zeiss Auriga Cross Beam equipped with a secondary electron detector. For sample preparation, the powder samples were dispersed on silicon wafers and coated with a 15 nm chromium layer using a Q150T Quorum pumped coater to enhance surface conductivity. Additionally, transmission electron microscopy (TEM) was conducted using a JEOL JEM-2100Plis microscope. N_2_ and CO_2_ sorption studies were conducted using a Micrometrics 3Flex Adsorption Analyzer, following overnight degassing of the samples at 150 °C under vacuum and *in situ* degassing at 130 °C for 3 h. CO_2_ sorption was performed at 30 °C. Surface area calculations were performed using the Brunauer–Emmett–Teller (BET) equation, and pore size distribution was analyzed using the Barrett–Joyner–Halenda (BJH) equation.

To investigate the mode of charge separation between the two semiconductors, photocatalytic deposition of platinum was conducted using dihydrogen hexachloroplatinate(iv) hexahydrate in anhydrous isopropanol. The distribution of the deposited platinum was examined through SEM and energy dispersive X-ray (EDX) spectroscopy. A dispersion of the photocatalyst, at a concentration of 1 mg mL^−1^ in anhydrous isopropanol, was prepared and exposed to illumination with a 300 W Xe source from LOT-Quantum Design, equipped with an AM1.5G filter set to 1 sun (100 mW cm^−2^) intensity.

### Photocatalytic performance

Photocatalytic CO_2_ reduction experiments were carried out in a 20 mL gas-tight stainless steel photoreactor, equipped with a quartz window. The sample of interest was dispersed in anhydrous isopropanol and drop-cast onto a 32 mm diameter Cytiva Whatman™ quartz filter paper. This was subsequently dried on a hot plate for 30 min at 80 °C to ensure complete removal of the residual solvent. The filter was then placed inside the photoreactor, accompanied by 40 μL of distilled H_2_O drops positioned on the side opposite the sample. The photoreactor was quickly evacuated from air and then refilled with CO_2_. Subsequently, the system was purged with research-grade CO_2_ at a flow rate of 10 mL min^−1^ for 15 min. Both the inlet and outlet of the reactor were then sealed and left for 15 min to achieve equilibrium. The CO_2_ reduction experiments were conducted in batch mode for 1 h, illuminated with a 300 W Xe source from LOT-Quantum Design. This source was equipped with an AM1.5G filter and adjusted to simulate 1 sun (100 mW cm^−2^) intensity. All reactions were performed under ambient conditions of temperature and pressure. The amount of water, reaction time, and sample preparation method were optimized in our previous study that used pure bulk g-C_3_N_4_ and the same measurement system.^15^ After completing the reaction, the gases produced were analyzed using a Shimadzu QP 2020NX gas chromatograph (GC) unit, which was equipped with a barrier ionization discharge (BID) detector and a mass spectrometer (MS). The apparent quantum efficiency of the catalyst was determined by illuminating the sample with a 365 nm LED light. To validate the source of CO_2_, control experiments were conducted under various conditions: (1) in helium (He) with H_2_O, (2) in He without H_2_O or CO_2_, (3) in the absence of a catalyst (using only quartz filter paper), and (4) in the dark. Isotope-labeled experiments were also carried out with ^13^CO_2_ instead of ^12^CO_2_. The symbol ± denotes the standard error.

## Results and discussion

**Fig. 1:**
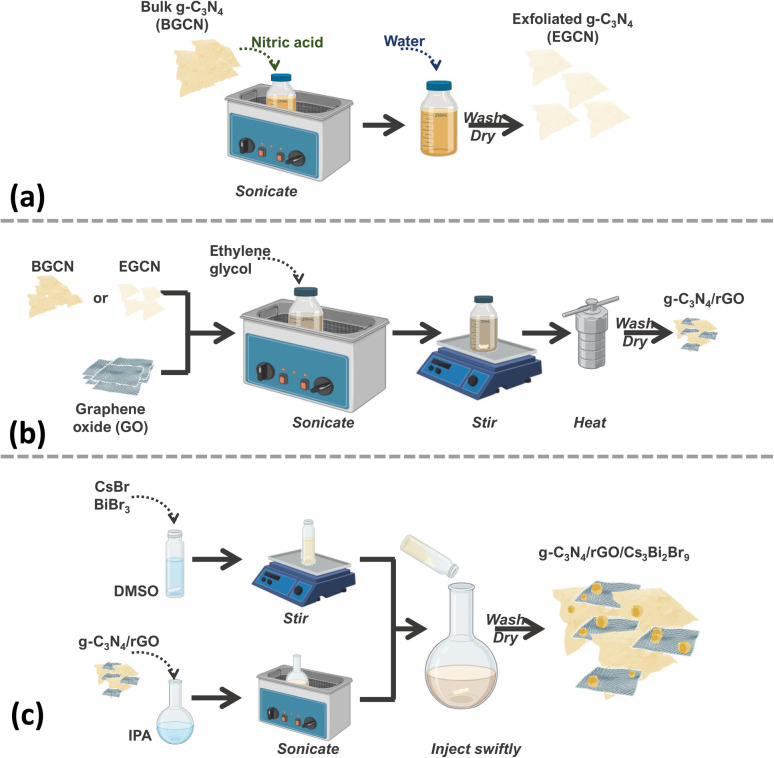
Schematic representation of the synthesis process of (a) exfoliated GCN, (b) GCN/rGO, and (c) GCN/rGO/CBB.

The synthesis of the final composite photocatalysts of g-C_3_N_4_/rGO/Cs_3_Bi_2_Br_9_ (GCN/rGO/CBB) followed the optimization of the g-C_3_N_4_ (GCN) exfoliation process and the rGO addition to GCN. As depicted in Fig. 1, exfoliated g-C_3_N_4_ (EGCN) was synthesized using a straightforward acid treatment procedure. Three samples of EGCN were produced by varying the ultrasonication time (0.5, 2, or 4 h). The sample with the highest photoactivity was selected for stage (b) where different quantities of graphene oxide (GO, 1, 2, or 5%.) were reduced on the surface to produce EGCN/rGO samples. Finally, an anti-solvent crystallization process was performed to crystallize Cs_3_Bi_2_Br_9_ on the surface of EGCN/rGO, resulting in the EGCN/rGO/CBB composite. The same optimization procedure was also performed on bulk g-C_3_N_4_ (BGCN) to produce the BGCN/rGO/CBB composite.

### Exfoliation of GCN

BGCN underwent nitric acid and sonication for different time intervals (0.5, 2, 4 h) to yield 3 different samples of EGCN. X-ray diffraction (XRD) of BGCN showed a small and broad peak at 13° (2*θ*) and a pronounced graphitic peak at 27.6° (2*θ*) corresponding to the (100) and (002) planes respectively (Fig. S1†).^32^ Upon acid exfoliation, the backbone structure of GCN shows slight changes: both XRD peaks showed a decrease in intensity, in agreement with a reduction in correlation length.^33^ In addition to this, a slight shift in the (002) peak to higher angles (to 27.8°) was observed post-treatment, which indicates changes to the main graphitic chain due to possible oxidation.^34^ The BGCN and EGCN samples were further characterized using FT-IR spectroscopy (Fig. S1†). BGCN had an intense and broad vibration centered at 3126 cm^−1^ assigned to N–H and O–H stretching. Sharp peaks detected within 1635–1444 cm^−1^ and 1392–1240 cm^−1^ are assigned to C=N and aromatic C–N stretching vibrational modes, respectively. A sharp peak at around 805 cm^−1^ is assigned to the tri-*s*-triazine ring vibrations.^32,35^ Upon acid exfoliation, the intensity of the absorption peak centered at 3126 cm^−1^ increases indicating more N–H or O–H stretching vibrations.

**Fig. 2:**
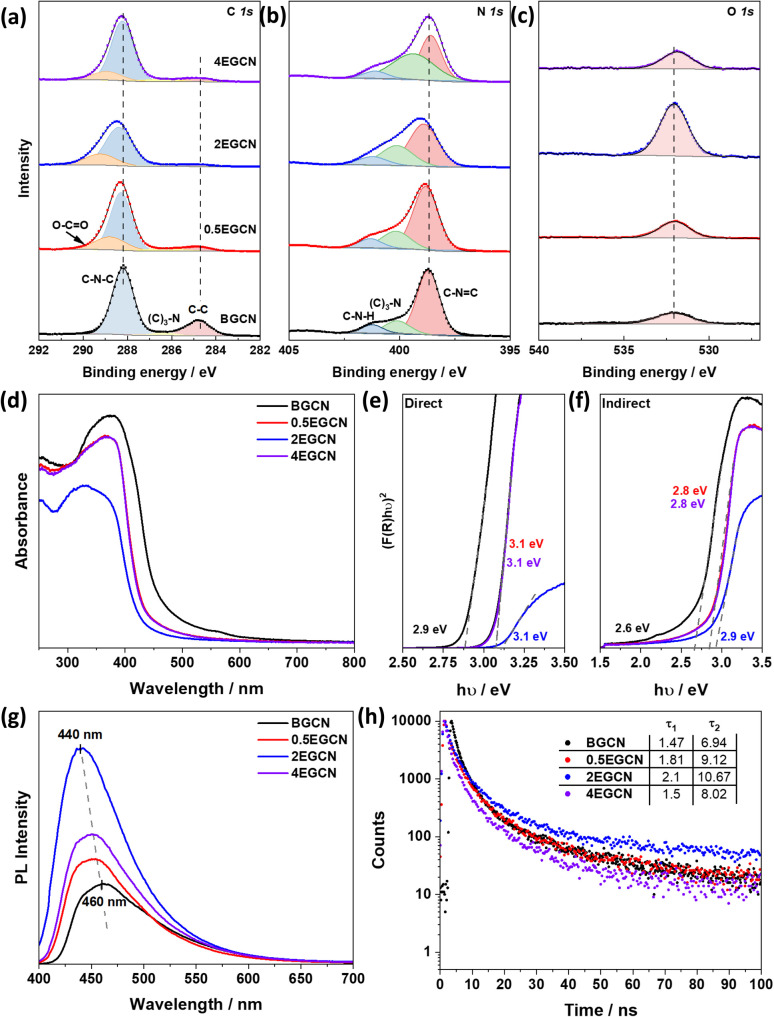
XPS (a) C 1s, (b) N 1s, and (c) O 1s scans of BGCN and EGCN samples. (d) UV-Vis absorbance spectra and calculated Tauc plots showing (e) direct and (f) indirect band gap extrapolation for BGCN and EGCN samples. (g) Steady-state PL spectra of the samples taken at a laser excitation wavelength of *λ*_ex_ = 380 nm. (h) Time-resolved PL taken at an excitation wavelength of *λ*_ex_ = 405 nm.

To better understand the effect of exfoliation on the surface composition of GCN, XPS was performed on the samples (Fig. 2a–c). High-resolution spectra of C 1s and N 1s peaks were recorded to characterize the backbone composition of GCN while O 1s spectra were used to determine any changes in surface oxidation. The C 1s scan of BGCN was deconvoluted into three bands at binding energies (BEs) of 284.8, 296.4, and 288.2 eV ascribed to C–C (or C=C), (C)_2_–N, and N–C=N bonds within the aromatic rings, respectively. Upon acid treatment, an additional band appeared at a BE of 288.8 eV assigned to the formation of O–C=O groups at the surface. This band was most intense after 2 h of acid exfoliation with a 23.3% area for the peak ascribed to O–C=O compared to 22 and 14.2% for 0.5EGCN and 4EGCN, respectively. All samples showed similar peaks in the N 1s scans that could be deconvoluted into three peaks centered at 398.7, 400.1, and 401.2 eV representing pyridinic C=N–C, graphitic (C)_3_–N, and pyrrolic H–N–C bonding, respectively.^36^ The intensity of the N 1s peaks for the 2EGCN sample was lower with respect to BGCN, indicating the possible replacement of surface nitrogen atoms with oxygen. Based on the XPS spectra, the atomic percentage of oxygen was 3.0, 3.6, 11.1, and 3.7% for BGCN, 0.5EGCN, 2EGCN, and 4EGCN samples.

UV-vis absorption spectra of the as-prepared BGCN compared to the exfoliated EGCN ones are shown in Fig. 2d. A distinct blueshift in the absorption edge of GCN was observed upon exfoliation. The change is attributed to quantum confinement effects and provides further evidence of changes in the morphology to thinner, sheet-like materials. Band gap calculations, as illustrated in Fig. 2e and f, revealed an increase in the direct band gap from 2.9 eV (BGCN) to a maximum of 3.1 eV (2EGCN) and in the indirect band gap from 2.6 eV (BGCN) to a maximum of 2.9 eV (2EGCN).^37^ In agreement with the larger optical band gap, upon acid exfoliation the PL peaks showed an increase in intensity and a blue shift from 460 nm for BGCN to 440 nm for 2EGCN, the sample with the highest level of surface oxidation (Fig. 2g).^37^ Transient PL decay measurements conducted on the BGCN and EGCN samples showed an increase in charge carrier lifetime upon acid exfoliation, reaching maximum with 2EGCN (Fig. 2h).^38^

**Fig. 3:**
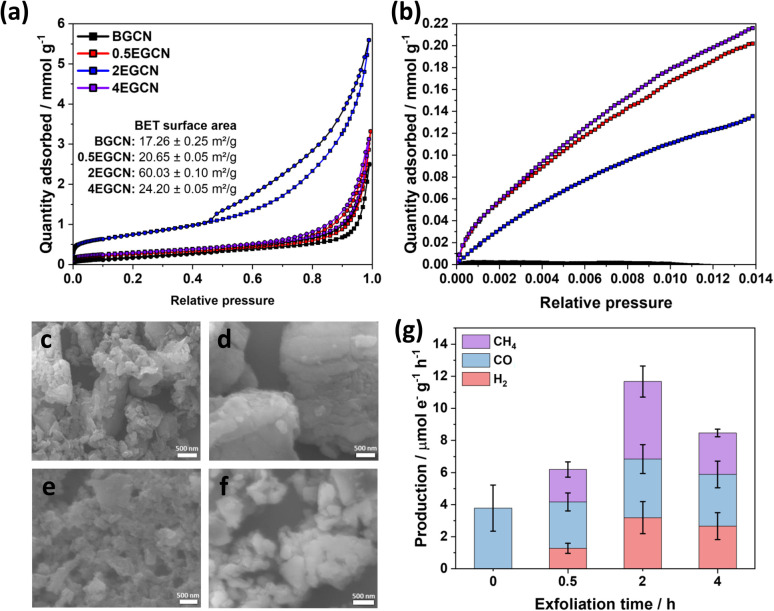
(a) N_2_ and (b) CO_2_ adsorption (□)/desorption (○) isotherms for bulk and exfoliated GCN with SEM micrographs of (c) BGCN, (d) 0.5EGCN, (e) 2EGCN, and (f) 4EGCN. (g) Production rates of the bulk and exfoliated GCN samples tested in the gas-phase under 1 sun illumination for 1-h batch reactions.

To understand the effect of acid exfoliation on the surface area of the bulk and exfoliated GCN samples, N_2_ sorption tests were performed, and the surface area was calculated using the Brunauer–Emmett–Teller (BET) model (Fig. 3a). All GCN samples exhibited adsorption and desorption at high pressures indicative of nitrogen sorption on the material surface and in interstitial voids within non-rigid aggregates.^39–41^ Notably, the 2-h exfoliated GCN (2EGCN) showed additional adsorption at low relative pressure, suggesting the presence of micropores. This sample also demonstrated strong hysteresis, indicative of small platelet-like structures forming ink-bottle-shaped pores with narrow necks.^42^ The BET surface area calculations revealed the highest surface area at 60.0 m^2^ g^−1^ for the 2EGCN sample, approximately 3.5 times larger than bulk GCN (Fig. 3a inset). Carbon dioxide (CO_2_) sorption studies at room temperature can be used to track any changes in the surface active sites of the catalyst upon treatment. The results indicated a significant increase in CO_2_ adsorption for the EGCN samples compared to BGCN (Fig. 3b). The increase in oxidation had a clear effect on the number of active sites for CO_2_ sorption on GCN possibly due to the introduction of pores and surface defects that had increased the overall surface area as well as the addition of functional groups that play a role in the physisorption of CO_2_ on the surface.^43,44^ SEM confirmed these changes in surface area, with all GCN samples displaying an inhomogeneous and amorphous morphology. BGCN showed irregular clusters averaging 400 nm in size (Fig. 3c–f). Acid exfoliation fragmented these clusters into thin platelets, particularly evident in the 2EGCN sample, which displayed an average size of 100 nm and minimal aggregation, correlating well with its higher BET surface area.

Gas-phase photocatalytic testing of the bulk and exfoliated GCN samples was conducted using CO_2_ gas and water vapor from a 40 μL H_2_O drop in a stainless steel photoreactor illuminated with simulated sunlight at 100 mW cm^−2^. A series of 1-h batch reactions were conducted on the samples after drop casting them onto a *Ø*25 mm quartz filter. As shown in Fig. 3g, BGCN produced 3.8 (±1.4) μmol_e^−^ CO_ g^−1^ h^−1^. Upon 0.5 h of acid exfoliation, the production of CO decreased to 2.9 (±0.6) μmol_e^−^ CO_ g^−1^ h^−1^, but 1.28 (±0.3) μmol_e^−^ H_2__ g^−1^ h^−1^ and 2.1 (±0.5) μmol_e^−^ CH_4__ g^−1^ h^−1^ evolved, which raised the total production on an electron basis from 3.8 (±1.4) to 6.2 (±0.8) μmol_e^−^_ g^−1^ h^−1^. When the exfoliation time was increased to 2 h the production of CO was similar to that of BGCN with 3.7 (±0.9) μmol_e^−^ CO_ g^−1^ h^−1^, but it also produced 3.2 (±0.9) μmol_e^−^ H_2__ g^−1^ h^−1^ and 4.8 (±1.0) μmol_e^−^ CH_4__ g^−1^ h^−1^. The total production of 2EGCN was 11.7 (±1.7) μmol_e^−^_ g^−1^ h^−1^. Upon 4 h of exfoliation, the total production decreased back slightly to 8.5 (±1.2) μmol_e^−^_ g^−1^ h^−1^. Overall, optimal exfoliation increases the production of CO and results in co-production of H_2_ and CH_4_. Consequently, 2EGCN was selected for all subsequent experiments based on these results. A breakdown of all production rates for H_2_, CO, and CH_4_ in μmol g^−1^ h^−1^ is summarized in Table S1.†

### Incorporation of reduced graphene oxide (rGO)

**Fig. 4:**
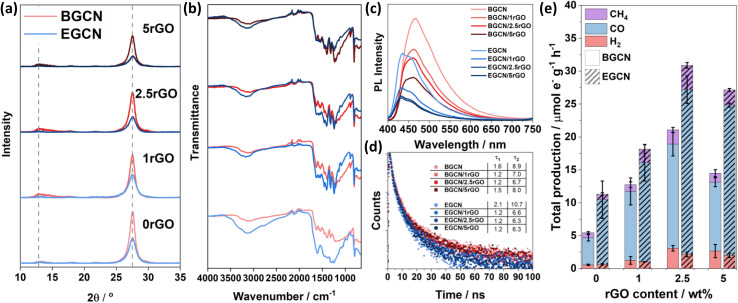
(a) XRD diffractograms and (b) FT-IR spectra of BGCN/rGO and EGCN/rGO samples at different rGO wt%. (c) Steady-state PL spectra recorded using a laser excitation wavelength of *λ*_ex_ = 380 nm and (d) time-resolved PL taken at an excitation wavelength of *λ*_ex_ = 405 nm for BGCN/rGO and EGCN/rGO samples. (e) Production rates of the BGCN/rGO and EGCN/rGO samples for H_2_, CO, and CH_4_ on an electron basis.

A hydrothermal treatment procedure was used to reduce set weights of graphene oxide (GO) on the surface of BGCN and 2EGCN as base samples for the synthesis. XRD diffractograms showed no additional peaks to the characteristic GCN peaks at 13 and 27.6° (2*θ*) upon the addition of rGO (Fig. 4a). However, compared to the pristine GCN samples, the peak at 13° (2*θ*) broadened upon the incorporation of rGO. In addition, the graphitic peak at 27.6° (2*θ*) decreased in intensity with the increase in rGO content suggesting the presence of lattice defects (rGO). The changes in XRD also reflect a slight reduction in the oxygen groups on the surface of BGCN and EGCN upon hydrothermal treatment. These observations could be further confirmed with a decrease in the –OH peak at around 3200 cm^−1^ in FT-IR spectra (Fig. 4b). Steady-state PL spectra presented in Fig. 4c for BGCN and BGCN/rGO at different rGO wt% showed a similar emission as the ones previously without rGO (Fig. 2d) with a broad peak centered at 465 nm. A clear decrease in the intensity of the PL peak was observed with the increase in rGO wt%. On the other hand, the main EGCN/rGO peak showed a blue shift in comparison to BGCN/rGO peaks to around 435 nm. However, the hydrothermal process used to reduce rGO led to a slight reduction of EGCN surface groups. For this reason, the peak centered at 454 nm, previously observed in BGCN samples, was detected. A similar decrease in intensity was observed with the addition of rGO. In both cases, the quenching is an indication of enhanced charge transfer in the composite in comparison to the pure GCN samples. To further validate the observation, transient PL was conducted on the samples and the calculated charge lifetimes are summarized in Fig. 4d. Among the different composites, BGCN/2.5rGO and EGCN/2.5rGO demonstrated the lowest lifetimes suggesting the most efficient charge separation. Upon the addition of rGO to the surface of BGCN, an almost four-fold increase in the overall photocatalytic production was observed from 5.5 (±0.5) to 21.1 (±1.9) μmol_e^−^_ g^−1^ h^−1^ with 2.5 wt% rGO (Fig. 4e). For EGCN, the highest production was also observed when 2.5 wt% rGO was added to reach 30.9 (±2.2) μmol_e^−^_ g^−1^ h^−1^ with a selectivity of 81% towards CO. A full breakdown of the production rates for H_2_, CO, and CH_4_ is summarized in Fig. S2.†

### Synthesis of GCN/rGO/CBB

**Fig. 5:**
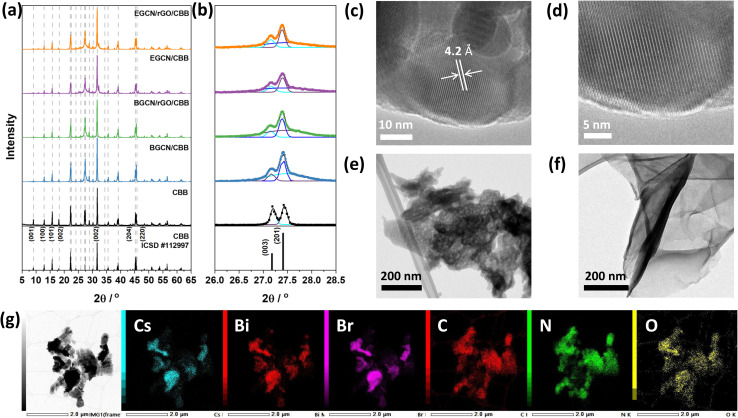
(a) X-ray diffractograms of CBB composites and (b) a zoomed-in section of the diffractograms. (c and d) TEM micrographs of CBB crystals, (e) 2EGCN, and (f) rGO sheets. (g) TEM micrographs of EGCN/rGO/CBB with EDX mapping of Cs, Bi, Br, C, N, and O elements.

Following the optimization of GCN exfoliation time and rGO content, GCN/rGO/CBB composites were formed with BGCN/2.5rGO and EGCN/2.5rGO by dispersing them in anhydrous isopropanol and inducing the antisolvent crystallization of Cs_3_Bi_2_Br_9_ (CBB). X-ray diffractograms of the as-prepared CBB crystals (Fig. 5a and b) displayed a pattern that represents the trigonal *P*3̄*m*1 space group (ICSD #112997).^45^ The displayed peaks proved the crystalline nature of the semiconductor with peaks at 13.1, 16.1, 22.4, 27.2, 27.4, 31.9, 35.8, 39.4, and 45.5° (2*θ*) corresponding to the planes (100), (101), (102), (003), (201), (202), (212), and (220), respectively.^15,46^ The addition of BGCN, EGCN, BGCN/rGO, and EGCN/rGO did not largely affect the crystalline structure of CBB. However, taking a closer look at the region between 26 and 28.5° (2*θ*) where the (003) and (201) peaks of CBB coincide with the (002) peak of GCN, a deconvolution showed that the ratio of the CBB peaks had changed suggesting a suppressed preferential growth in the presence of a secondary material during the synthesis (Fig. 5b). The observed diffraction patterns of CBB confirm the crystalline nature of the semiconductor as opposed to the amorphous structures of both GCN and rGO. As can be observed in the TEM micrographs of pure CBB (Fig. 5c and d), the crystals were hexagonal in shape with a lattice spacing of approximately 4.2 Å corresponding to the (102) plane and similar to previously reported values in the literature.^15,47^ In contrast, TEM micrographs of BGCN (Fig. S3†) and 2EGCN (Fig. 5e) powders did not show any crystalline lattice. In comparison to pure BGCN, 2EGCN also had irregularly shaped clusters but were smaller in size and showed clear signs of pores within the structure (Fig. S3†). Micrographs of rGO proved their wrinkly, sheet-like and amorphous structure as expected based on the literature (Fig. 5f).^48,49^ When rGO, CBB, and 2EGCN were combined, clusters on CBB were observed to form and disperse on the organic sections of the composite as demonstrated in the TEM micrograph and EDX mapping in Fig. 5g.

**Fig. 6:**
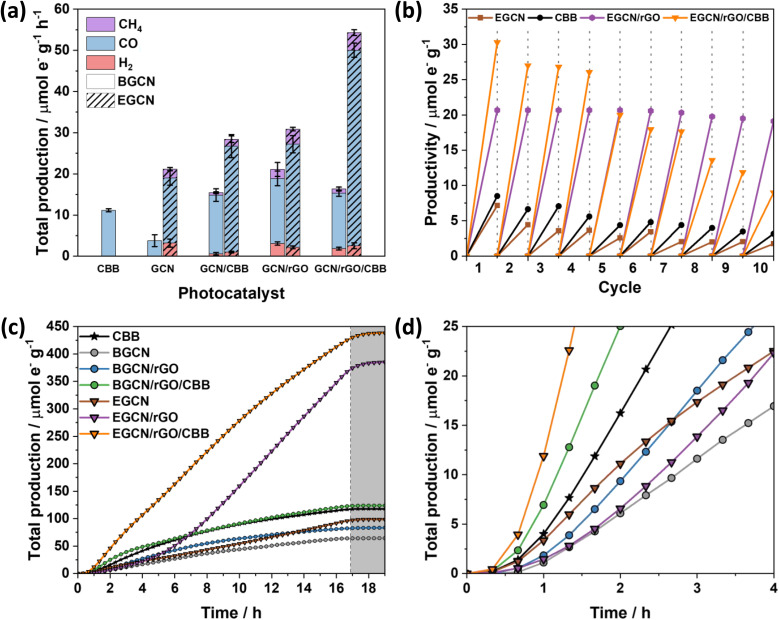
(a) Total production of H_2_, CO, and CH_4_ of BGCN and EGCN samples coupled with only rGO, only CBB, and rGO with CBB on an electron basis. (b) Recyclability experiments performed at 1 h intervals for 10 cycles on pure CBB, EGCN, EGCN/rGO, and EGCN/rGO/CBB. (c) Total production on an electron basis for 16 h continuous flow reactions performed at 0.5 mL min^−1^. (d) Zoomed-in plot of the total production on an electron basis for the first 4 h of the reaction. All the reactions were performed under 1 sun, using CO_2_ gas and water vapors.

As displayed in Fig. 6a, the photocatalytic activity of pure CBB on an electron basis was 11.2 (±0.3) μmol_e^−^_ g^−1^ h^−1^. Growing CBB crystals on BGCN and EGCN through antisolvent crystallization showed an improvement in photocatalytic production by 2.5 and 1.4 times compared to pure BGCN and EGCN, respectively. Even though BGCN/rGO/CBB showed a 1.5 and 3 times increase in activity compared to pure CBB and BGCN, respectively, and the final composite did not demonstrate a significant change compared to BGCN/CBB or BGCN/rGO. In contrast, the EGCN/rGO/CBB composite displayed superior photocatalytic activity to all the other samples with 54.3 (±1.9) μmol_e^−^_ g^−1^ h^−1^ and an apparent quantum efficiency (AQE) of 0.001% when tested using a 365 nm monochromated light source at an intensity of 100 mW cm^2^. Control tests performed on EGCN/rGO/CBB in the absence of CO_2_, H_2_O, a photocatalyst, or light were conducted (Fig. S4†). The results showed less than 10% production in the absence of one or both reactants formed from adventitious carbon on the surface and no production in the absence of the photocatalyst or light verifying the photocatalytic properties of the catalyst. Furthermore, isotope labelled experiments using ^13^CO_2_ were conducted on the best sample, EGCN/rGO/CBB, under the same reaction conditions. The spectra in Fig. S5† show a clear peak at an *m*/*z* of 29 assigned to the production of ^29^CO from ^13^CO_2_. Table S2† lists the photocatalytic activity of EGCN/rGO/CBB, and other composites prepared in the literature, together with their different reaction conditions (*e.g.*, light used).^14,50–56^ EGCN/rGO/CBB shows very competitive results.

Repeatability tests were performed on the best sample, EGCN/rGO/CBB, and its constituents EGCN, CBB, and EGCN/rGO over ten 1 h cycles (Fig. 6b). Pure CBB and EGCN both demonstrated a decline in photoactivity after the first cycle and lost around 50% of their production by the fifth cycle. Overall, after 10 cycles both samples lost 75% of their production. In contrast, EGCN/rGO maintained its activity across the 10 cycles losing only 10% of their overall activity. We hypothesize that the incorporation of rGO helped improve the stability of EGCN due to better charge transport to the rGO and a decrease in electron accumulation on the surface of EGCN causing degradation. EGCN/rGO/CBB also shows excellent stability, significantly better than pure CBB with a slight decrease in activity by the 5^th^ cycle (25% loss). However, after 10 cycles, the photoactivity of the composite decreased to 9.8 μmol_e^−^_ g^−1^ h^−1^ with an overall 75% loss.

To delve into the rates of photocatalytic production, continuous flow reactions were performed on the pure samples as well as the composites. The accumulated total production (μmol_e^−^_ g^−1^) of CO, H_2_ and CH_4_*vs.* time is represented in Fig. 6c and d. EGCN/rGO/CBB outperformed all the other samples accumulating approximately 438.3 μmol_e^−^_ g^−1^ over 16 h of irradiation, in accordance with the batch reaction results. The samples displayed different initial rates of production, *i.e.*, slopes (Fig. 6c). Pure BGCN and EGCN samples displayed a similar trend where the number of accumulated products increased steadily with time and an inflection point was reached at around 2 h after which it began to decline and plateau. The addition of rGO in BGCN/rGO and EGCN/rGO moved the inflection point to around 6–8 h. In contrast to BGCN/rGO, EGCN/rGO did not decline after the inflection point but increased and maintained a relatively constant rate of production until the irradiation was stopped at 16 h (like in EGCN/rGO). However, between the 6th and the 7th hour of the reaction, a change in the rate of production was observed. This observation could be linked to the cycling tests previously discussed in Fig. 6b when the production of the EGCN/rGO/CBB samples began to decline. Overall, the composite demonstrated superior photocatalytic activity compared to the other samples over the 16-h reaction time. Pure CBB and all composites containing CBB demonstrated a large initial rate of production that was maintained for at least 4 h (Fig. 6d). In the case of EGCN/rGO/CBB, we observe the benefits of all rGO, EGCN, and CBB. There is a large initial rate of production in the first hours (like in all CBB samples) and a large rate of production is maintained for 16 h (like in EGCN/rGO). The production only plateaued when the light was switched off after 16 h. rGO and CBB added to EGCN synergistically improved the overall stability of the composite while maintaining a high production rate.

To understand the stability of the EGCN/rGO/CBB samples, the composite was characterized after a 1 h of reaction under 1 sun using XRD, FT-IR, and XPS. The results were compared to those of the fresh sample (Fig. S6†). Based on the XRD diffractograms shown in Fig. S6a,† the intensity of the peaks decreased slightly after the reaction indicating a slight deterioration in the crystal structure of CBB. In addition, FT-IR spectra showed a slight increase in surface oxidation of EGCN after the reaction (Fig. S6b†). Finally, the samples were analyzed using XPS before and after the reaction (Fig. S7†). Scans of C 1s, N 1s, and O 1s showed a slight increase in surface oxidation (as indicated in FT-IR) without any other significant changes indicating that the organic part of the composite (rGO and EGCN) did not deteriorate during the reaction. This result explains the stability of EGCN/rGO during the cycling tests (Fig. 6b). On the other hand, Bi 4f scans showed an insignificant decrease in overall area but a slight increase in the intensity of the Bi^0^ peak with respect to Bi^3+^ after the reaction. This was proof of the reduction of Bi^3+^ of Cs_3_Bi_2_Br_9_ upon light exposure leading to the deterioration in activity after several hours.

### Mechanism

Based on the reported photocatalytic test results, the system with 2.5 wt% rGO formed on EGCN (treated for 2 h) and combined with 40 wt% CBB outperformed all other catalyst systems. To understand the mechanism of charge transfer within this system, a combination of Kelvin probe and APS measurements were performed to determine the work function (*φ*) and valence band edge (*E*_v_) of the individual semiconductors, respectively (Fig. S8†). Using the direct band gap calculated for 2EGCN and CBB based on the UV-Vis DRS measurements, the conduction band edge (*E*_c_) of the semiconductors was located and the final energy level values are summarized in Table S3.†

**Fig. 7:**
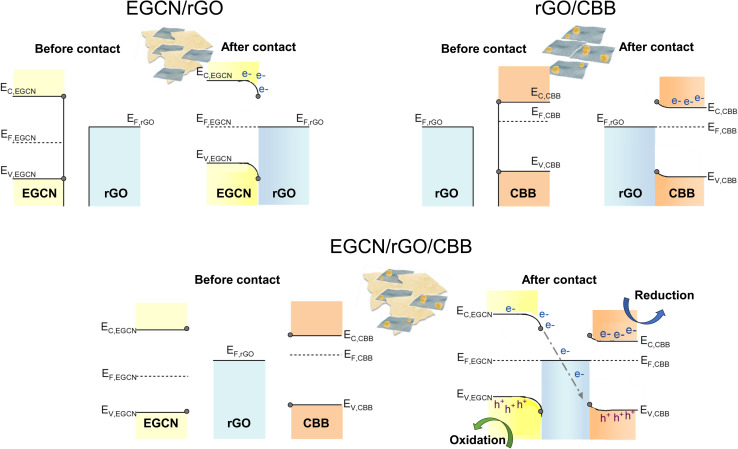
Schematic representation of charge transfer routes in the best performing system with rGO, EGCN, and CBB.

The energy levels (*E*_c_, *E*_F_, and *E*_v_) of EGCN, CBB, and rGO and the band bending expected at their different interfaces are presented in Fig. 7. In the case of EGCN/rGO, since *E*_F,rGO_ was shallower than *E*_F,EGCN_, the equilibration of Fermi levels upon contact would induce downward band bending (*i.e.*, the electric field) in the EGCN that directs photoinduced electrons towards the rGO. This improved charge separation explains the improvement in photocatalytic production with EGCN/rGO samples. In contrast, when rGO and CBB come into contact, the Fermi level equilibration would result in an upward band bending in CBB because its *E*_F_ is shallower than that of rGO. This band bending also improves charge separation by helping with the transport of photogenerated holes towards the rGO and the electron accumulation in the conduction band of CBB. Both interfacial band bendings explain the improved photocatalytic activity of EGCN/rGO/CBB: The photogenerated electrons move towards the rGO where they can recombine with the holes transferred from CBB. Simultaneously, photoinduced electrons in CBB can accumulate in its conduction band while holes photogenerated in EGCN can accumulate in its valence band (Fig. 7 bottom). This charge transfer mechanism is known as a mediated Z-scheme between EGCN and CBB where photoinduced charges are spatially separated with reduction taking place in CBB, oxidation in the EGCN, and redox charge mediation in the rGO.^57^ A mediated Z-scheme improves charge separation and ensures keeping the strongest drive for redox reactions with shallow electrons and deep holes.

**Fig. 8:**
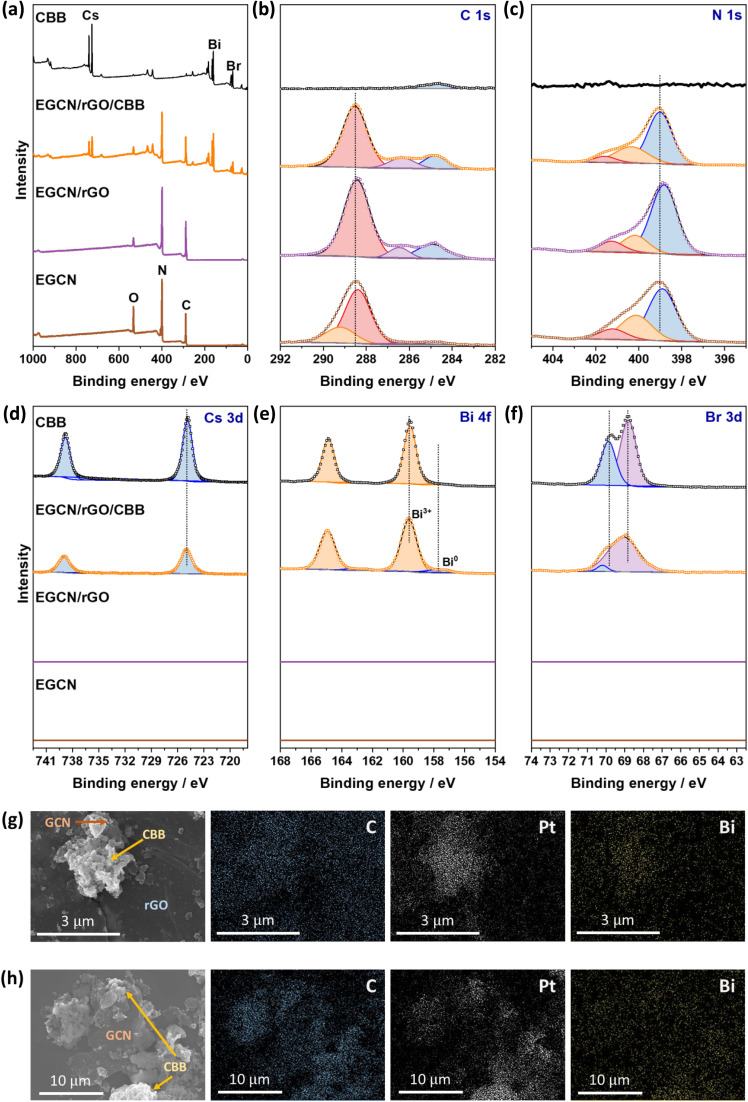
XPS (a) survey scans and high-resolution (b) C 1s, (c) N 1s, (d) Cs 3d, (e) Bi 4f, and (f) Br 3d spectra of EGCN, EGCN/rGO, EGCN/rGO/CBB, and CBB. SEM micrographs of the EGCN/rGO/CBB sample after Pt photodeposition along with EDX mapping of carbon, platinum, and bismuth elements for (g) an area with rGO, CBB, and EGCN and (h) an area with a high concentration of CBB and EGCN.

XPS spectra were collected for Cs 3d, Bi 4f, Br 3d as well as C 1s and N 1s of pure EGCN, EGCN/rGO, EGCN/rGO/CBB, and pure CBB ( Fig. 8). Cs 3d scans of both the pure CBB and its composite revealed a doublet at 724.9 and 738.8 eV ascribed to Cs 3d_5/2_ and Cs 3d_3/2_, respectively. Additionally, Br 3d scans of CBB and the composite showed the formation of two single peaks at 68.8 and 69.9 eV for Br 3d_5/2_ and Br 3d_3/2_, respectively. The Bi 4f scan of CBB depicted a doublet ascribed to Bi^3+^ species centered at 159.6 and 164.8 eV. While EGCN/rGO/CBB showed the same Bi^3+^ doublet, it also showed a secondary, low-intensity doublet at 157.8 and 162.9 eV indicating the presence of metallic Bi (0) elements in the composite. The formation of this Bi (0) indicates a possible excess of electrons on the surface of CBB when in contact with EGCN/rGO that leads to reduction of Bi^3+^ cations on the surface, in agreement with the band bending analysis in Fig. 7. Finally, a comparison between the C 1s and N 1s of pure EGCN and EGCN/rGO/CBB showed a slight shift to higher binding energy and suggests a decrease in electron density on the surface of EGCN upon the construction of the composite which confirms possible downward band bending at the EGCN/rGO interface, as previously represented in the energy band construction (Fig. 7). A summary of the elemental peaks for XPS is presented in Table S4.† Overall, the XPS results demonstrated the accumulation of electrons on the surface of CBB as well as a downward bend bending of EGCN which, coupled with the proposed mechanism in Fig. 7, indicates the formation of a mediated Z-scheme heterojunction.

Furthermore, the mechanism of photocatalytic CO_2_ reduction was confirmed by conducting *in situ* photocatalytic reduction of a Pt salt and analyzing where Pt deposits. EGCN/rGO/CBB powder was dispersed in anhydrous isopropanol in the presence of dihydrogen hexachloroplatinate(iv) hexahydrate. The dispersion was illuminated using the same light conditions used for the photocatalytic CO_2_ reduction reactions. After 1 h, the powder was collected and washed with anhydrous isopropanol to remove any residue of the unreacted Pt precursor. The sample elements were then mapped using EDX to identify the location of Pt deposition within the sample. Fig. 8g shows an area within the sample with all three constituents present: CBB, rGO, and EGCN. Observing the morphology of the SEM micrograph, CBB was located in clusters on EGCN. EDX mapping of the elements showed a high density of Pt deposition on the semiconductor clusters rather than on the rGO signifying that electrons were not accumulating on this sheet-like material and that it was used as an electron mediator and not as a reduction site. To understand the flow of electrons, a second mapping area was chosen showing EGCN and CBB clusters in particular (Fig. 8h). The respective mapping of the area showed a higher density of Pt deposition in areas with a high concentration of CBB (crystals shown in the SEM micrograph of Fig. 8h). The preferential reduction of Pt on CBB indicated that it was indeed the semiconductor with a higher concentration of electrons. The direction of electron transfer was then from the EGCN to CBB with rGO acting as a mediator, which confirms the mediated Z-scheme mode of charge transfer as indicated in the schematic in Fig. 7.

## Conclusions

This study underscores the significant role of photocatalytic CO_2_ reduction in the advancement of solar fuels, emphasizing the critical importance of catalyst efficiency for sustainable energy production. We have achieved notable improvements in the performance of graphitic carbon nitride (g-C_3_N_4_) as a photocatalyst through surface modifications, such as exfoliation and surface oxidation. The strategic integration of reduced graphene oxide (rGO) in optimized ratios has been particularly effective in mitigating charge recombination, leading to a significant enhancement in photocatalytic activity. A key advancement of this research is the development of a hybrid inorganic/organic heterojunction combining g-C_3_N_4_/rGO with Cs_3_Bi_2_Br_9_, culminating in the g-C_3_N_4_/rGO/Cs_3_Bi_2_Br_9_ composite. This composite has shown superior performance over both pure Cs_3_Bi_2_Br_9_ and bulk g-C_3_N_4_, achieving an overall production rate of CO, H_2_, and CH_4_ from CO_2_ and H_2_O of 54.3 (±2.0) μmol_e^−^_ g^−1^ h^−1^. The energy diagram constructed for this system indicates that rGO serves as an effective redox mediator within a Z-scheme heterojunction, facilitating efficient reduction and oxidation processes. The successful development of this high-performance heterojunction composite not only showcases its potential as an effective photocatalyst for CO_2_ reduction but also marks a significant stride towards realizing the potential of solar fuels in sustainable energy solutions.

## Data availability

The data that support the findings of this study are openly available in a research data repository at https://doi.org/10.6084/m9.figshare.25896976.

## Author contributions

Yasmine Baghdadi: conceptualization; formal analysis; investigation; methodology; validation; writing. Matyas Daboczi: investigation. Filipp Temerov: investigation. Mengya Yang: investigation. Junyi Cui: investigation. Salvador Eslava: conceptualization; funding acquisition; supervision; writing – review & editing.

## Conflicts of interest

There are no conflicts to declare.

## Supplementary Material

TA-012-D4TA01857E-s001
