# Supplementary material for: A g-C3N4/rGO/Cs3Bi2Br9 mediated Z-scheme heterojunction for enhanced photocatalytic CO2 reduction
Source: J Mater Chem A Mater. 2024 May 28;12(27):16383–95. doi: 10.1039/d4ta01857e (PMC11232668; doi:10.1039/d4ta01857e)
Supplement: TA-012-D4TA01857E-s001 [file TA-012-D4TA01857E-s001.pdf]

Electronic Supplementary Material (ESI) for Journal of Materials Chemistry A.
This journal is © The Royal Society of Chemistry 2024

Supporting Information

g-C_3N_4/rGO/$Cs_3Bi_2Br_9$ mediated Z-scheme heterojunction for enhanced photocatalytic CO_2 reduction

Yasmine Baghdadi [a], *Matyas Daboczi* [a], *Filipp Temerov* [a, b], *Mengya Yang* [a], *Junyi Cui* [a], *Salvador Eslava* *[a]

[a] Department of Chemical Engineering, Imperial College London, London SW7 2AZ, United Kingdom.

[b] Nano and molecular system (NANOMO) research unit, University of Oulu, Oulu 90570, Finland.

E-mail: s.eslava@imperial.ac.uk

Apparent quantum yield calculations

To measure the apparent quantum yield (AQE), the sample was tested in the same experimental setup using the same conditions while replacing the 300 W Xe lamp with a 365 nm monochromatic LED lamp. To perform the calculations, the following parameters were used:

Irradiance,	I	= 100 mW cm^{-2} = 1000 W m^{-2}
Area of effective light irradiation,	A	= $8x10^{-4}$ m^2
Plank's constant,	h	$= 6.626x10^{-34}$ J s
Speed of light,	c	$= 3x10^{17}$ nm s^{-1}
Wavelength of incident light,	λ	= 365 nm
Avogadro's number,	N_A	$= 6.022x10^{23}$ mol^{-1}

Incident light intensity, (I_0) = IxA

Photon energy, (E_p) = $\frac{hc}{\lambda} = \frac{(6.626x10^{-34} J\ s)x(3x10^{17}\ nm\ s^{-1})}{365\ nm}$

Therefore, the number of incident photons per unit time (N_P) can be calculated as:

$$N_P = \frac{I_0}{E_P}$$

The number of moles of incident photons per unit time (M_P) can be calculated by dividing N_P by N_A such that:

$$M_P = \frac{N_P}{N_A}$$

Finally, AQE is calculated by dividing the moles of reacted electrons by M_P such that:

$$AQE\ (\%) = \frac{2xH_{2produced} + 2xCO_{produced} + 8xCH_{4produced}}{M_P} x100$$

Supporting data

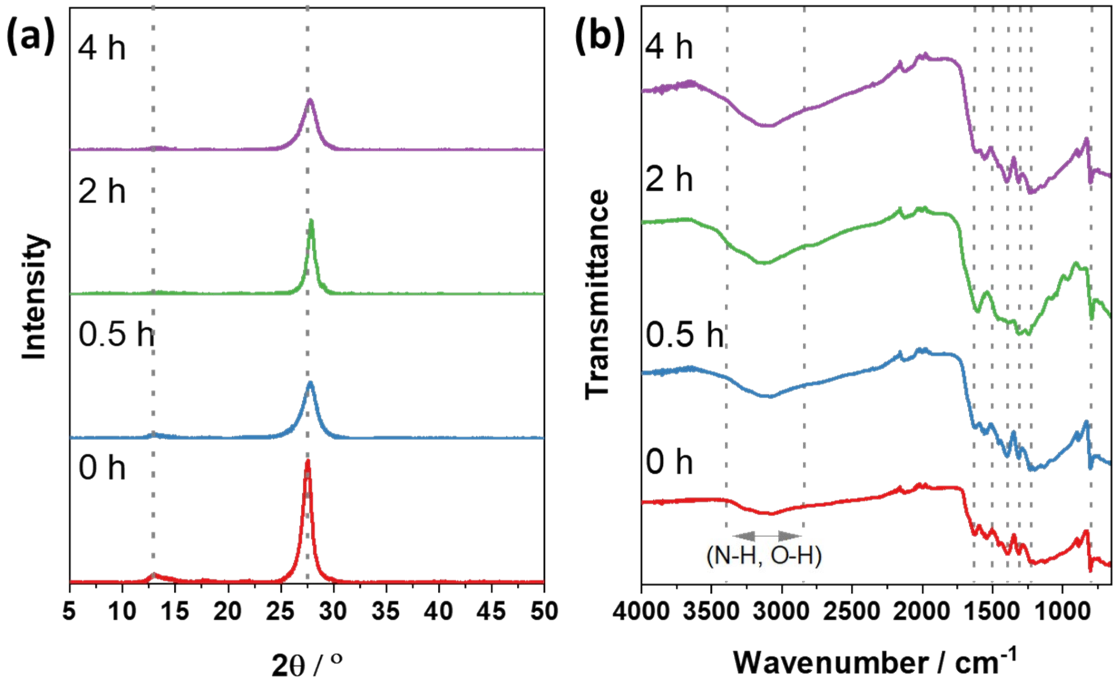

Figure S1: (a) X-ray diffractograms and (b) FT-IR spectra of BGCN (0 h) and EGCN (0.5, 2, 4 h) samples.

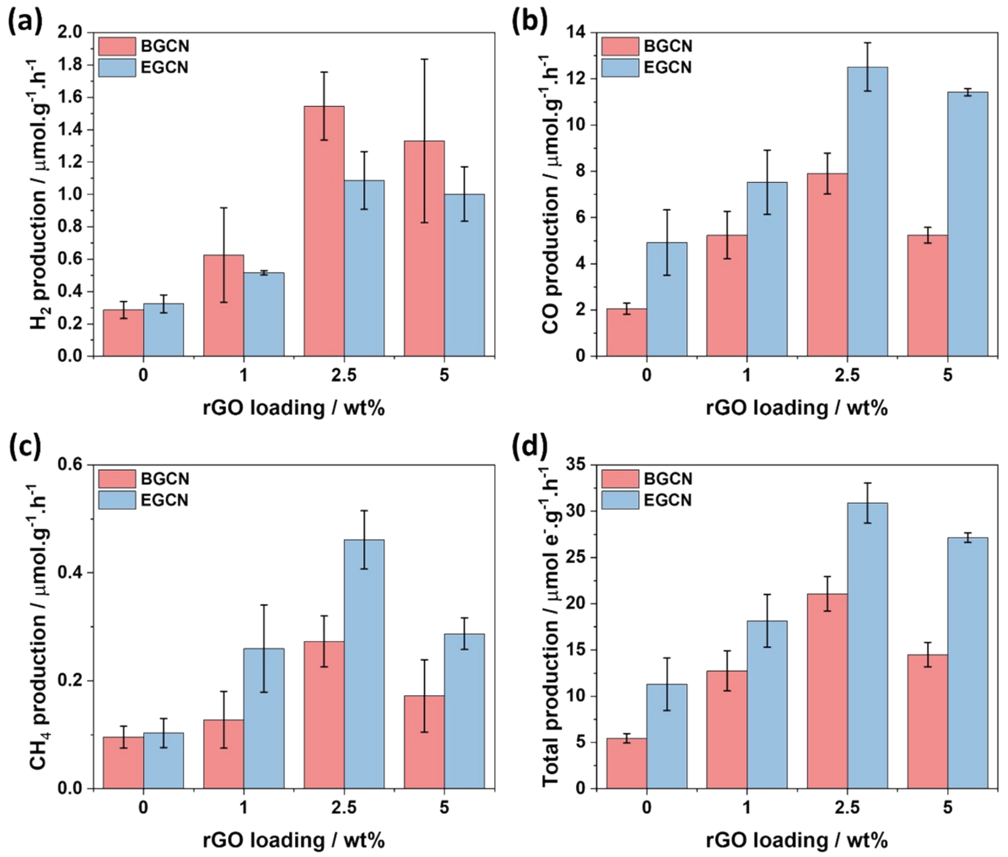

Figure S2: Production rates of the BGCN/rGO and EGCN/rGO samples for (a) H_2, (b) CO, and (c) CH_4. (d) Total production on an electron basis.

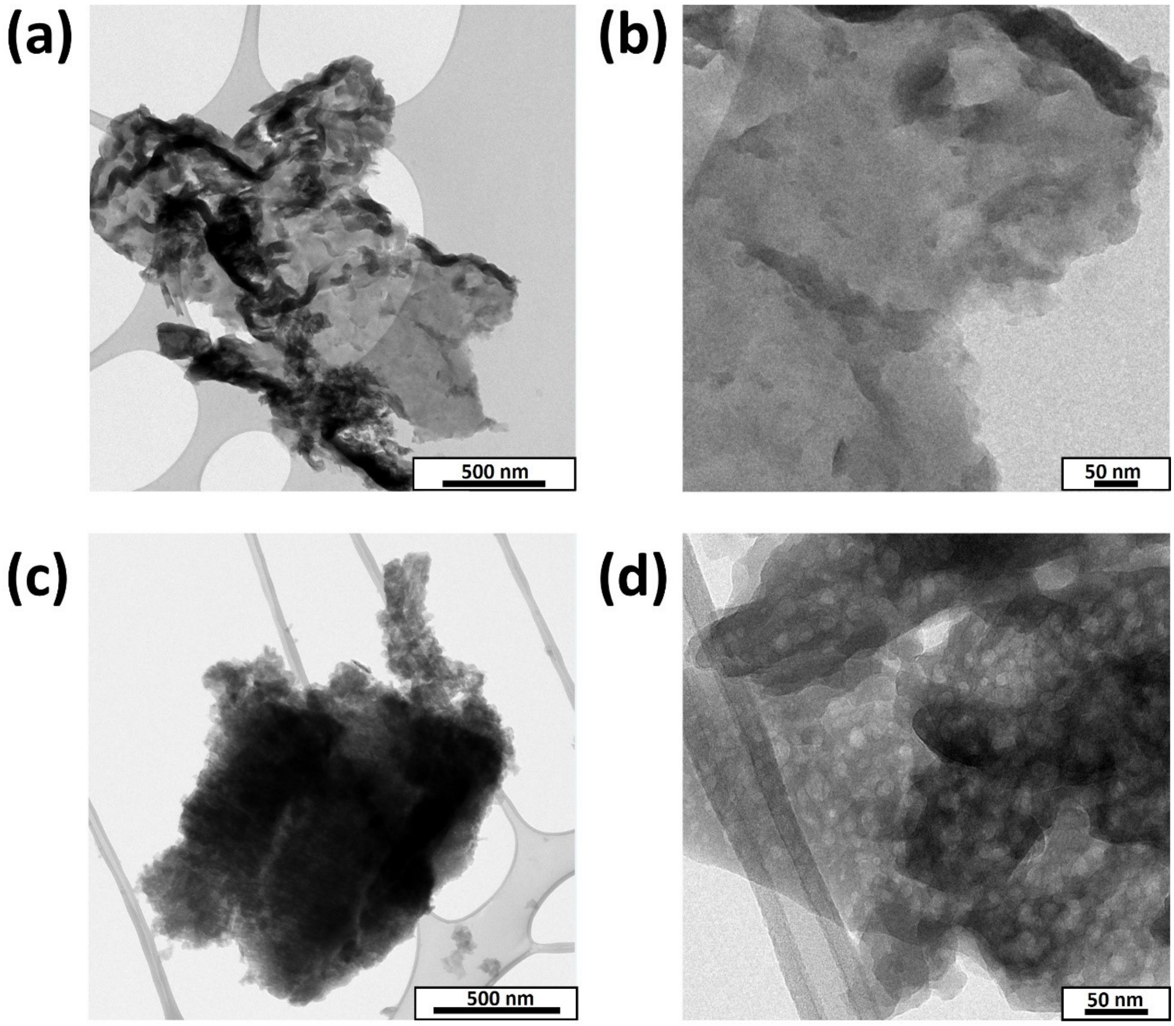

Figure S3: TEM micrographs of (a-b) BGCN and (c-d) 2EGCN.

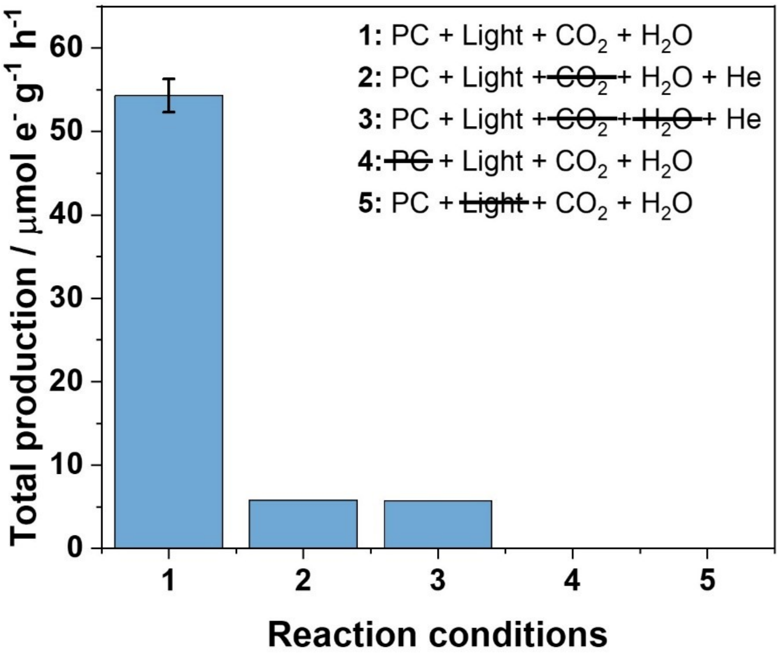

Figure S4: Control tests performed on EGCN/rGO/CBB in the absence of (2) CO_2, (3) CO_2 and H_2O, (4) the photocatalyst, and (5) light.

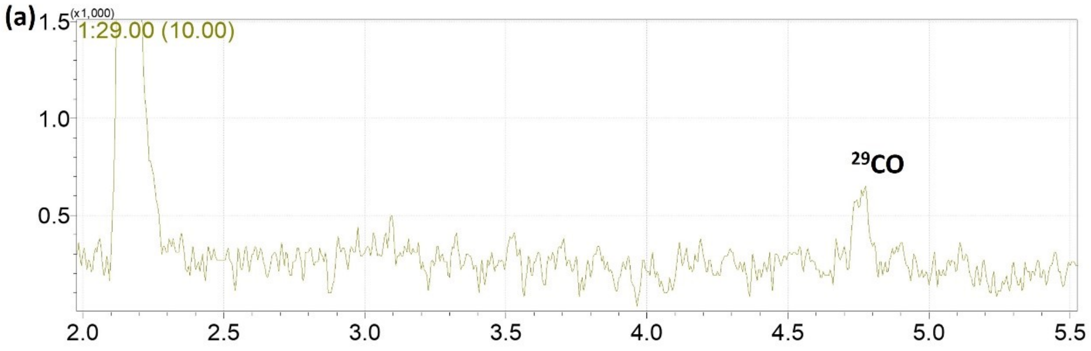

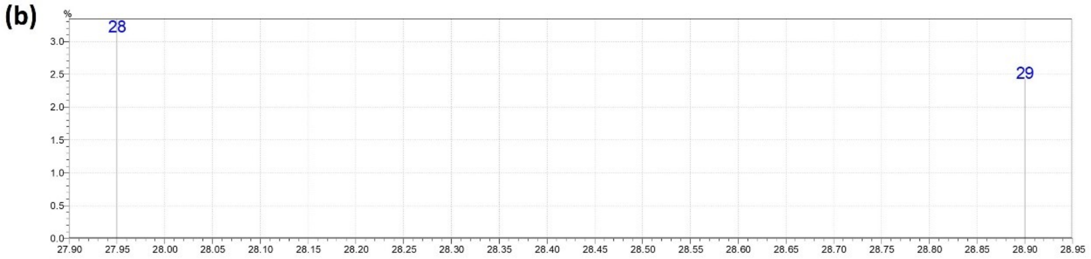

Figure S5: Mass chromatography spectra showing (a) fragmented peaks for m/z 28 and 29 with retention time and (a) relative intensity with respect to m/z ratio after conducting a 2 h batch reaction on EGCN/rGO/CBB using isotope labelled $^{13}CO_2$

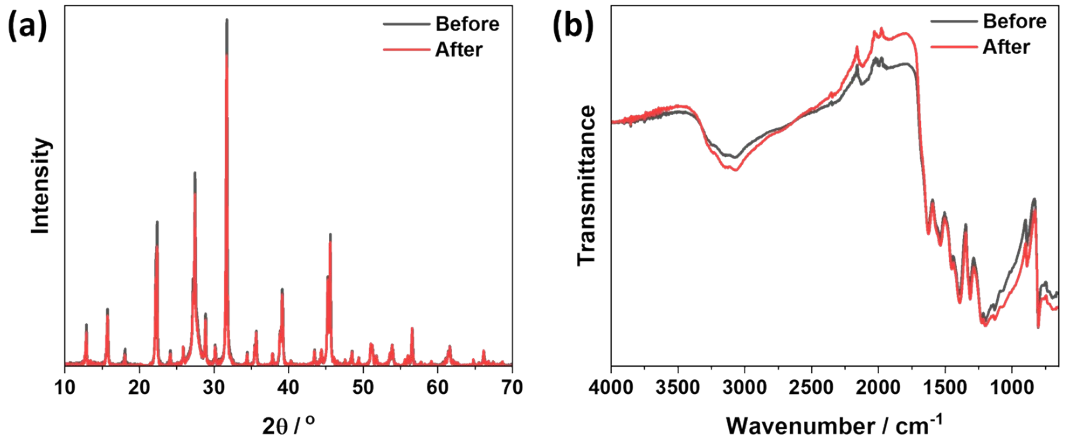

Figure S6: (a) XRD diffractograms and (b) FT-IR spectra of EGCN/rGO/CBB sample before and after a 1 hr reaction.

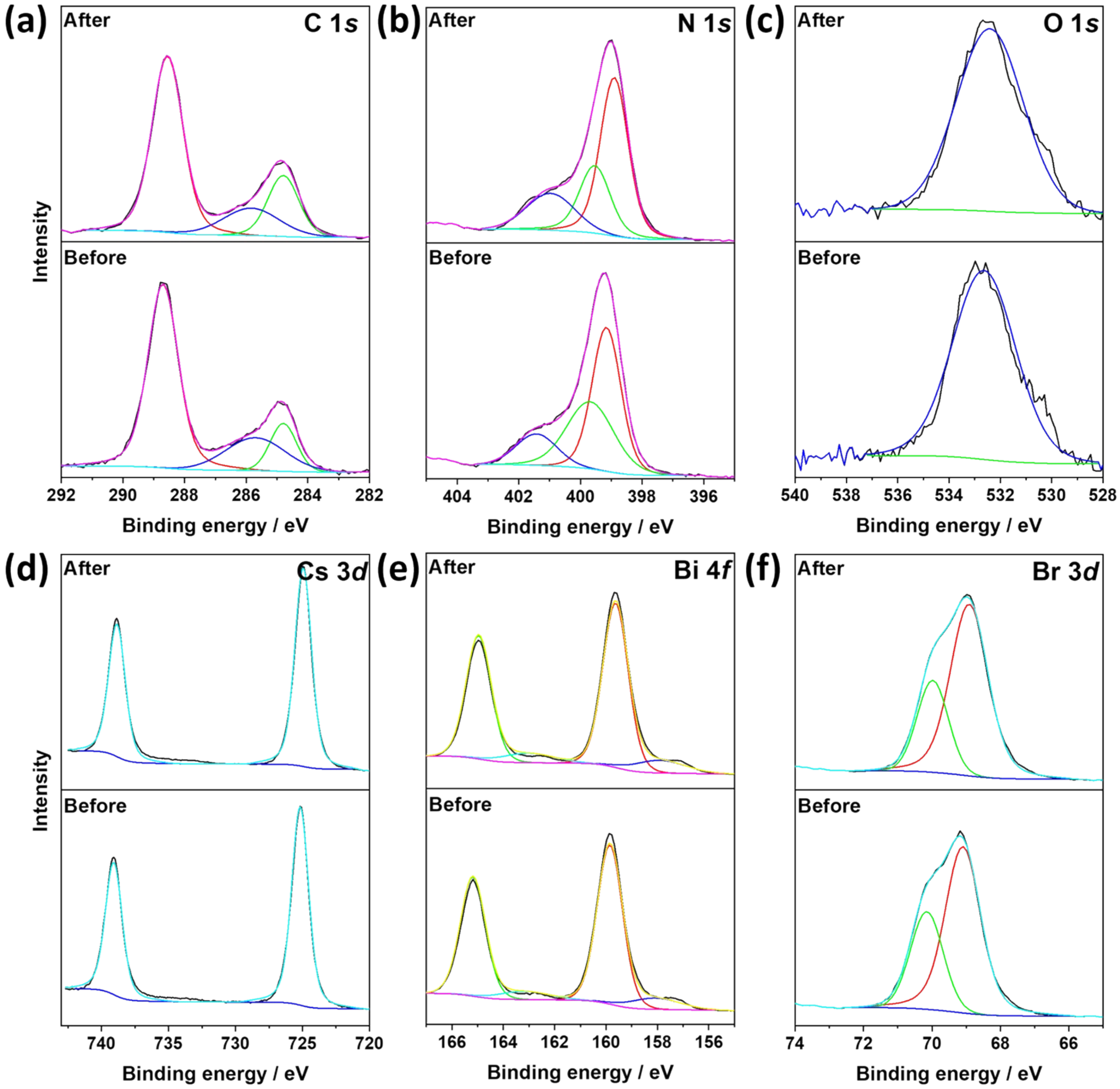

Figure S7: XPS scans of (a) C 1*s*, (b) N 1*s*, (c) O 1*s*, (d) Cs 3*d*, (e) Bi 4*f*, (f) Br 3*d* for EGCN/rGO/CBB samples before and after a 1 hr reaction.

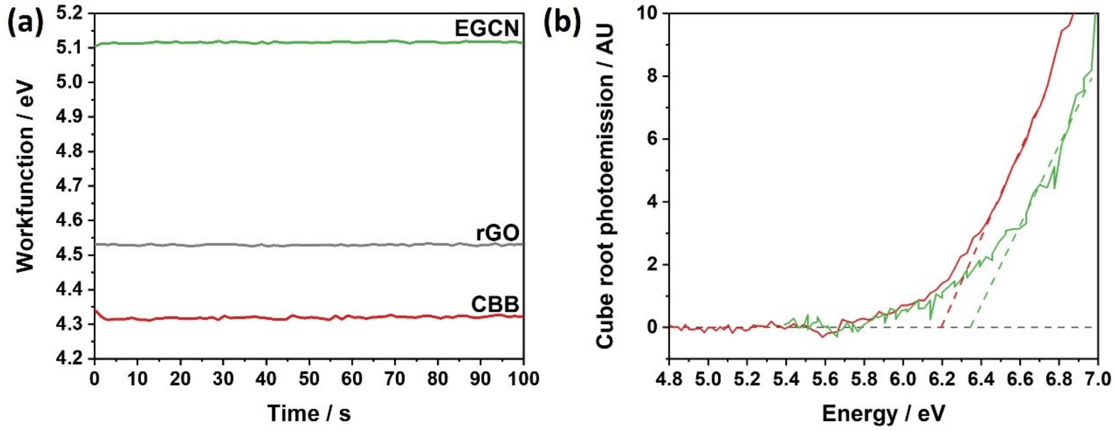

Figure S8: (a) Work function values of rGO, 2EGCN, and CBB from Kelvin probe measurements. (b) Cube root photoemission data extrapolated to gather valence band edge positions of 2EGCN and CBB.

Table S1: Summary of production rates of H_2, CO, and CH_4 for all samples in µmol $g^{-1}h^{-1}$

	Production (µmol $g^{-1}h^{-1}$)		
	H_2	CO	CH_4
**BGCN**	0.00 ± 0.00	2.06 ± 0.24	0.00 ± 0.00
**0.5EGCN**	0.64 ± 0.16	1.45 ± 0.28	0.25 ± 0.06
**2EGCN**	1.59 ± 0.50	1.83 ± 0.45	0.60 ± 0.12
**4EGCN**	1.33 ± 0.42	1.61 ± 0.42	0.32 ± 0.03
**BGCN/1rGO**	0.63 ± 0.29	5.24 ± 1.03	0.13 ± 0.05
**BGCN/2.5rGO**	1.55 ± 0.21	7.90 ± 0.89	0.27 ± 0.05
**BGCN/5rGO**	1.33 ± 0.51	5.23 ± 0.34	0.17 ± 0.07
**EGCN/1rGO**	0.52 ± 0.01	7.53 ± 1.39	0.26 ± 0.08
**EGCN/2.5rGO**	1.09 ± 0.18	12.51 ± 1.05	0.46 ± 0.05
**EGCN/5rGO**	1.00 ± 0.17	11.42 ± 0.15	0.29 ± 0.03
**BGCN/CBB**	0.29 ± 0.16	7.13 ± 0.76	0.07 ± 0.02
**BGCN/rGO/CBB**	0.92 ± 0.17	6.72 ± 0.36	0.13 ± 0.06
**EGCN/CBB**	0.50 ± 0.11	12.88 ± 1.40	0.21 ± 0.10
**EGCN/rGO/CBB**	1.27 ± 0.35	23.76 ± 0.86	0.53 ± 0.09

Table S2: Summary of similar photocatalytic systems reported in literature for photocatalytic CO_2 reduction.

Photocatalyst	Production (µmol $g^{-1}h^{-1}$)		Medium	Reaction conditions	Light source	Ref. in manuscript
g-C_3N_4/rGO/$Cs_3Bi_2Br_9$	H_2	1.3	CO_2 (g) saturated with H_2O	ambient conditions	300 W Xe, AM 1.5G, 100 mW cm^{-2}	This work
	CO	23.8				
	CH_4	0.5				
40 wt% $Cs_3Bi_2Br_9$/g-C_3N_4	CO	14.2	CO_2 (g) saturated with H_2O	ambient conditions	300 W Xe, AM 1.5G, 100 mW cm^{-2}	[1]
MCM-41/$Cs_3Bi_2Br_9$	CO	17.2	CO_2 (g) saturated with H_2O	20 ℃	300 W Xe ($\lambda \geq$ 420 nm), 350 mW cm^{-2}	[2]
g-C_3N_4/rGO/NiAl-LDHs	CO	2.6	CH_3CN:TEOA:H_2O = 3:1:1 (v/v/v)	10 ℃	300 W Xe, 1000 mW cm^{-2}	[3]
	CH_4	20.0				
g-C_3N_4/NiAl-LDHs	CO	8.2	CO_2 (g) saturated with H_2O	ambient conditions	300 W Xe ($\lambda \geq$ 420 nm)	[4]
g-C_3N_4/rGO	H_2	68	0.2 M $NaHCO_3$ solution saturated with CO_2	ambient conditions	300 W Xe, AM 1.5G	[5]
	CH_3OH	114				
rGO with g-C_3N_4/CdS	CO	23.93	TEOA/H_2O solution saturated with CO_2	0.4 MPa	300 W Xe	[6]
$CsPbBr_3$/g-C_3N_4	CO	28.5	CO_2 (g) saturated with H_2O	-	300 W Xe ($\lambda \geq$ 420 nm)	[7]
$Cs_3Bi_2Br_9$/$BiVO_4$	CO	70.63	CO_2 (g) with H_2O	25 °C, 101.3 kPa	300 W Xe, AM 1.5G, 100 mW cm^{-2}	[8]

Table S3: Summary of the band gap, Work function, and band edge positions of the materials

Material	Band gap (E_g) / eV	Conduction band (E_C) / eV	Work function (φ) / eV	Valence band (E_V) / eV
**GO**	-	-	-4.588	-
**rGO**	-	-	-4.526	-
**BGCN**	2.9	-3.52	-5.056	-6.39
**EGCN**	3.1	-3.37	-5.116	-6.48
**CBB**	2.6	-3.59	-4.319	-6.19

Table S4: Summary of XPS elemental peaks for CBB, EGCN, EGCN/rGO, and EGCN/rGO/CBB

		Peak binding energy (eV)			
		**EGCN**	**EGCN/rGO**	**EGCN/rGO/CBB**	**CBB**
**C 1*s***	C-C	284.80	284.80	284.80	284.80
	$(C)_3$-N	286.28	286.35	286.34	-
	C-N-C	288.39	288.29	288.53	-
	O-C=O	289.21	-	-	-
**N 1*s***	C-N=C	298.90	398.82	399.01	-
	$(C)_3$-N	400.11	400.17	400.34	-
	C-N-H	401.22	401.25	401.59	-
**Cs**	3*d*5	-	-	724.97	724.91
	3*d*3	-	-	738.90	738.83
**Bi^0**	4*f*7	-	-	157.82	-
	4*f*5	-	-	162.93	-
**Bi^{3+}**	4*f*7	-	-	159.62	159.54
	4*f*5	-	-	164.93	164.80
**Br**	$3d_{5/2}$	-	-	69.08	68.83
	$3d_{3/2}$	-	-	70.19	69.87

References

1. Baghdadi, Y., et al., *Cs3Bi2Br9/g-C3N4 Direct Z-Scheme Heterojunction for Enhanced Photocatalytic Reduction of CO2 to CO.* Chemistry of Materials, 2023. **35**(20): p. 8607-8620.
2. Cui, Z., et al., *Space-confined growth of lead-free halide perovskite Cs3Bi2Br9 in MCM-41 molecular sieve as an efficient photocatalyst for CO2 reduction at the gas–solid condition under visible light.* Applied Catalysis B: Environmental, 2022. **310**: p. 121375.
3. Zhou, D., et al., *Reduced graphene oxide assisted g-C3N4/rGO/NiAl-LDHs type II heterostructure with high performance photocatalytic CO2 reduction.* Chemical Engineering Journal, 2022. **450**: p. 138108.
4. Tonda, S., et al., *g-C3N4/NiAl-LDH 2D/2D Hybrid Heterojunction for High-Performance Photocatalytic Reduction of CO2 into Renewable Fuels.* ACS Applied Materials & Interfaces, 2018. **10**(3): p. 2667-2678.
5. Sahoo, R.C., et al., *Bandgap engineered g-C3N4 and its graphene composites for stable photoreduction of CO2 to methanol.* Carbon, 2022. **192**: p. 101-108.
6. Zhao, X., et al., *Reduced graphene oxide-modified Z-scheme g-C3N4/CdS photocatalyst with a staggered structure for the enhanced photoreduction of CO2.* Sustainable Energy & Fuels, 2022. **6**(16): p. 3768-3777.
7. Cheng, R., et al., *Incorporation of Cesium Lead Halide Perovskites into g-C3N4 for Photocatalytic CO2 Reduction.* ACS Omega, 2020. **5**(38): p. 24495-24503.
8. Zhou, B., et al., *Strain-Engineering of Mesoporous Cs(3) Bi(2) Br(9) /BiVO(4) S-Scheme Heterojunction for Efficient CO(2) Photoreduction.* Small, 2023. **19**(29): p. e2302058.
